# The capacity-building role of community liaison workers with refugee communities in Victoria, Australia

**DOI:** 10.1186/s13033-021-00485-9

**Published:** 2021-07-02

**Authors:** Karen Wei, Prem Chopra, Susie Strehlow, Mardi Stow, Ida Kaplan, Josef Szwarc, Harry Minas

**Affiliations:** 1 School of Population and Global Health, The University of Melbourne, Melbourne, Australia; 2 The Victorian Foundation for Survivors of Torture, Melbourne, Australia

**Keywords:** Capacity-building, Refugee mental health, Trauma-informed mental health, Community liaison workers

## Abstract

**Background:**

A range of services within Australia and internationally have been developed that are focused on the engagement of individuals who are of refugee background to work as a liaison between their communities and mental health services. The Community Liaison Worker (CLW) role at the Victorian Foundation for Survivors of Torture (VFST) was developed in 2008 in order to engage in such capacity-building initiatives.

**Aims:**

To review and document the establishment, evolution and current status of the VFST CLW role, and examine the perspectives of CLWs on their role in trauma-informed community capacity-building.

**Methods:**

The study comprised of two stages: a historical case study of the development of the CLW role, and a qualitative research study based on interviews with CLWs in order to identify key themes regarding various aspects of their role and understand the facilitators and barriers to their work of trauma-informed capacity-building with their respective communities.

**Results:**

The CLW role has evolved from the provision of direct services through joint work with Counsellor Advocates at VFST to a broader role that is focused on building the capacity of community members. Thematic analysis of interviews with the seven current CLWs identified the complexity of their dual role as members of their community and employees of VFST, their role in addressing short-term goals to meet community needs, and the long-term objective of empowering their community to become integrated and self-sufficient.

**Conclusions:**

CLWs at VFST demonstrate important work of liaison workers in facilitating trauma-informed capacity-building initiatives that are of benefit to members of their communities and also to service providers.

## Background

It is recognised that trauma-related disorders are highly prevalent in refugee communities, and that the nature and duration of persecution and torture are associated with the risk of trauma-related disorders [1]. There is significant heterogeneity in the reported prevalence of trauma-related disorders amongst refugees, with estimates of Post-traumatic stress disorder of up to 99%, anxiety disorders up to 88% and depression up to 100%, depending on the assessment criteria applied as well as the setting and population studied [1, 2]. Refugees often suffer multiple losses and potentially traumatic events, and the experience of torture in particular is recognised as a significant factor predisposing to adverse mental health effects [2].

Individuals of refugee background have substantial needs with regards to their physical and mental wellbeing, and yet it is recognised that they face significant challenges in seeking and engaging in appropriate treatment services [3]. Refugees face a number of barriers in accessing health care services, including language difficulties, lack of knowledge of mental health services, mistrust of services and authorities, economic challenges, and various cultural factors including stigma that may impact on their readiness to seek assistance, particularly with regards to mental illness [1, 3, 4]. Mainstream services themselves may not be culturally appropriate, and hence may not be effective in meeting the needs of people of diverse backgrounds, thereby reinforcing the stigma of mental illness [4].

In order to address these barriers in negotiating complex health systems, community capacity-building initiatives have taken the approach of empowerment by building the understanding of health as well as available services amongst community members themselves through local knowledge, skills and perspectives [5]. Not all capacity-building approaches are trauma-informed, and may include a range of strategies to address unmet needs identified by the community members themselves in areas such as housing, education and a range of social needs. Trauma-informed capacity-building approaches refer to initiatives that directly consider the needs of the community regarding their shared experience of trauma as well as their need for safety and collective healing [1, 5].

Members of refugee communities can play positive roles supporting the wellbeing of their communities through participation in trauma-informed capacity-building initiatives. While such roles are variously conceptualised and designated in the literature (e.g. ethnic cultural broker [6], bicultural worker [7, 8], refugee liaison [9], multicultural health broker [10, 11], community healthcare worker [11–13] and outreach support worker/bi-lingual support outreach community development worker [14]), central to all such roles is that liaison workers are members of a refugee community who are engaged in facilitating a liaison between the community and health, mental health and social services.

The literature demonstrates that liaison workers are able to facilitate access to a range of social services including financial [15] and employment services [6], educational institutions [9, 16], housing services [6, 15], legal services [6], faith-based communities [6], culturally-responsive health resources and services [8, 15], as well as supporting the development of networks between families with similar needs [17].

Liaison workers are a resource for mainstream service providers in their role as cultural consultants that extends beyond the role of interpreters [8, 15, 18]. Liaison workers are able to provide interpretation of various aspects of mainstream culture for refugees to assist them in their integration in community activities and to access services [6, 10, 16]. Liaison workers support access to existing services both within the organisation employing the liaison worker and also with external organisations [12, 19, 20].

Furthermore, as members of refugee communities themselves, liaison workers are an integral asset in developing community capacity-building programs that address the social determinants of health and other initiatives including peace building, health promotion, community leadership, and other related psychological and social wellbeing initiatives to empower refugees. Liaison workers are able to undertake these various roles through advocacy [14, 21], education and training [22–24], and as mentors [25, 26].

Trauma-informed care refers to an organisational framework that provides the context in which the impact of trauma is addressed at multiple levels in order to improve the outcomes of survivors [5]. This framework is founded on adherence to the following six principles: safety; trustworthiness and transparency; peer support; collaboration and mutuality; empowerment; voice and choice; cultural, historical and gender issues [5]. Trauma-informed capacity building is a process that seeks to strengthen and facilitate recovery of survivors and their community by strengthening relationships within the community and with external partners, developing community skills and resources, and enabling participation in decision-making and leadership [27, 28]. Such an approach acknowledges the capacity of refugees themselves to negotiate the challenges of settlement and to build on the strengths within their community [29]. There is a need to further understand the perspectives of liaison workers with respect to the contribution to their communities in trauma-informed capacity-building interventions.

The role of Community Liaison Workers (CLWs) within the Capacity Building (CaB) Team at the Victorian Foundation for the Survivors of Torture (VFST) is one such initiative in which members of refugee communities are engaged in trauma-informed capacity-building interventions. VFST was founded in 1987 with the mission of enhancing health and wellbeing, and protecting human rights, of people from refugee backgrounds who have experienced torture or other traumatic events. VFST delivers counselling and support services to survivors, and works together with government, service providers, the corporate sector as well as refugee background and broader communities to achieve its mission. VFST comprises of four integrated programs: (1) Direct services, (2) Community and Sector Development, (3) Practice Development and Learning, and (4) Human resources and administration operations [30].

### Aims

There were three aims of this research project. First, to review and document the establishment, evolution and current status of the VFST CLW role. Second, to describe the role of CLWs in trauma-informed capacity-building initiatives. Third, to examine the perspectives of CLWs on their role in trauma-informed community capacity-building.

## Methods

### Setting

The research was conducted on-site at the VFST office in Brunswick, Melbourne, between June 2018 and June 2019. During this 12 month period, VFST provided direct services to 4308 individuals (54% female and 46% male) from over 70 countries who were either permanent residents, people seeking asylum or temporary visa holders [31]. A range of interventions were provided encompassing individual and family work with children, adolescents and adults, group programs, psychiatric treatment provided through the refugee mental health clinic, and joint interventions with CLWs [31].

### Design

The study comprised two parts. An initial case study was conducted documenting the establishment, history and evolution of the CLW role, followed by a qualitative study exploring the perspectives of CLWs.

#### Part 1: Case study

The history and evolution of the CLW role between 2007 and 2019 was collated by the primary author based on a review of internal documentation, unpublished historical data, and interviews with three staff members at VFST involved in the establishment and ongoing development of the program.

#### Part 2: Qualitative study of the perspectives of CLWs

In order to understand the perspectives of CLWs regarding their contribution to communities in trauma-informed capacity-building interventions, the research utilised qualitative exploratory methods through seven individual in-depth interviews using a semi-structured interview guide.

### Study population

The study participants were individuals employed as CLWs at VFST [32]. At the time of the study, a diverse group of seven CLWs were employed at VFST from the Chin, Karen, Assyrian Chaldean, Syrian and Sierra Leonean refugee communities of Melbourne. All seven CLWs were invited by the primary author and agreed to participate.

### Data collection

Prior to the commencement of the research study, an interview guide was developed on the basis of a literature review regarding liaison workers in general, the Case Study report (Part 1), and consultations with staff members involved in the establishment of the program in order to identify key questions regarding the role of CLWs and the impact of capacity-building interventions on refugee communities.

Before commencement of interviews, participants were given information about the project (both orally and in the form of a written plain language statement). All interviews were recorded and later transcribed.

A brief demographic survey was also developed for use at the commencement of the interview to gather information about each participant including gender, age, highest academic qualification, duration of employment, languages used for CLW position, cultural identity, and average hours per week employed by VFST.

The primary author conducted all of the individual semi-structured interviews with the recruited participants. Interviews were carried out in English using semi-structured interviewing in a manner that was gender and culturally sensitive, ethical, and incorporating open-ended questions [33].

### Data analysis

Approval for the study was obtained through the VFST Institutional Ethics Committee (Identification number: 2018-01).

Audio-recordings of participant interviews were transcribed verbatim. Participants were identified using a unique participant code. All potentially identifying data were either de-identified or excluded in the analysis.

Transcribed Microsoft Word documents were stored on a password-protected media at VFST premises, as well as the password-protected personal computer at the primary author’s university during the data analysis phase. Transcribed data were deleted from the primary author’s computer upon completion of the research project. Interview transcripts and consent forms are being stored securely at VFST premises for five years and will then be destroyed.

Interview transcripts were analysed using thematic analysis from a deductive approach [34, 35]. The domains identified in the semi-structured interview guide provided a structure to categorise themes. In order to identify the views of participants directly, an emic perspective was adopted [34]. Themes identified were independently verified by the secondary author, followed by detailed discussion in order to refine the final themes and reach consensus. Subsequently the summation of themes was presented to other members of the research group who were not directly involved in the interview process or initial analysis for external verification.

## Results

### Part 1: Case study

#### History and evolution of the role of CLWs

Since the establishment of VFST in 1987 it was identified that there was a gap between the model of service provision through counselling and advocacy services, and the needs of the community as a whole with regards to their experience of trauma and their role in trauma recovery [27]. It was recognised that for a number of refugee communities, the idea of accessing trauma counselling and psychosocial support services was challenging and potentially stigmatising. As a result, it was observed that although assessment and support by VFST was undertaken on-arrival in Australia, the offer of ongoing counselling and support was frequently not taken up because of different conceptualisations and understandings of mental health and treatment [27].

In order to better understand and address the broader needs of their communities, a decision was made to employ CLWs. The CLW role was initially defined as a member of the refugee community, employed at VFST for the purpose of establishing a connection between members of their community and VFST. This initiative saw the employment of six individuals in 2007 for an initial period of six months. The composition of the CLW group was determined in response to the incoming populations under the Australian Government’s Refugee and Humanitarian Program at the time. This was made up of refugee minorities from Burma including the Chin community arriving from Malaysia and India, the Karen community arriving from camps on the Thai-Burma border [36], as well as refugees of Assyrian-Chaldean ethnicity from Iraq. Thus the initial CaB Team members belonged to the Chin, Karen and Assyrian-Chaldean communities, with two CLWs from each of these communities. Subsequently the Capacity Building (CaB) Team, consisting of the CLWs and a program coordinator, was formally established in 2008.

VFST’s capacity-building strategy is grounded in an ecological approach that is defined by a number of key principles, according to which all interventions:

1. address individual, family, community and society levels,
2. reflect community priorities,
3. implement prevention and treatment strategies,
4. prioritise building the capacity of people to gain control over resources to enhance lives, and
5. identify and build the strengths of community members through an active process of collaboration [37].

The changing profile of refugee communities influenced the work of VFST, and the engagement of CLWs from specific communities. The team over time grew to include CLWs of Sierra Leonean, Afghan, Sri Lankan Tamil, Hazara and Syrian backgrounds, although not all positions have been ongoing.

Counsellor Advocates at VFST are engaged within the Direct Services Program to provide individual, group and family support services through specialised trauma-informed counselling and advocacy work [30]. In the early stages, the CaB Team carried out the majority of their work with individuals and families who required counselling and advocacy. CLWs were engaged as “language and cultural support” workers in their joint work with Counsellor Advocates. VFST recognised the need to strengthen the capacity of the organisation to engage more effectively with these communities, develop the capacity of other services in the sector, and also build the capacity of community. Hence the CLW role soon evolved beyond its initial purpose, with the additional role of establishing relationships with members of their community, as well as between other organisations and VFST.

The CaB Team has adapted over time, responding to the changing make-up of refugee communities in Victoria. The CaB Team has worked within budgetary constraints to respond to the needs of new refugee arrivals, which has been influenced by global politics, international events and changes in Australia’s Humanitarian Program intake [38]. In addition, the needs of refugee communities themselves have emerged over time, shifting from initial settlement demands to ongoing challenges in the acculturation process. In response the CLW role has adapted to remain relevant to the community and has included a number of community-specific projects to address particular needs such as the ‘The Coming Together: Two Cultures, One Life’ project with members of the Sudanese community, the ‘The Supporting the Health and Wellbeing of Karen Youth’ project and the ‘Early Childhood Access and Participation’ project with the Chin community to name a few [27, 28].

Over time, the CLWs’ learning and knowledge of the impact of trauma, mental health, counselling and capacity-building has grown. According to the CaB Team Coordinator, a number of CLWs have undertaken personal and professional development courses and higher education degrees that have in turn enhanced their approach to capacity-building initiatives.

The CaB Team has also sought the contribution of others in particular roles, including a pro bono consultant with overseas experience as a Community Development Worker in South East Asia. For specific projects, the CaB Team has utilised interpreters to assist in language support where there are multiple dialects spoken in the one group. Whilst the CLW role is much broader, and encompasses a range of ongoing activities of relevance to trauma-informed capacity building, Bicultural Workers are also employed by VFST for the completion of specific projects over a defined contract period [27, 28].

The CaB Team Coordinator facilitates a weekly supervision session with CLWs to allow for exploration, discussion, and support in relation to a broad range of issues relating to their work in trauma-informed capacity-building.

As VFST services have expanded and grown, the organisation has opened regional offices across Melbourne. The CaB Team continues to be funded by an allocation from VFST’s Commonwealth government funding. Additional philanthropic funding has allowed the CaB Team to engage in other specific projects to name a few [27, 28].

#### Current status of CaB team

The overall goal of VFST’s CaB Team is to strengthen the capability of refugee communities in successfully managing the challenges of acculturation by supporting the development of social networks and through engagement with services to develop meaningful roles in Australian society [39]. Participation by communities themselves in identifying their priorities and issues in resettlement is fundamental to regaining control, building new connections and promoting trauma-informed recovery goals when rebuilding lives.

The current CaB Teamwork plan focuses on three streams of interventions: (1) joint work with Counsellor Advocates from the Direct Services Program, (2) group work, and (3) community capacity-building work.

The current practice of joint work with Counsellor Advocates includes the provision by CLWs of assistance with community engagement, cultural advice, interpreting and advocacy. CLWs assist in the development of trust in the counselling process, which can be culturally unfamiliar to community members. They also enhance the Counsellor Advocates’ understanding of the person’s cultural background and provide an insight into the experience of mental health from their community perspective [27].

The CaB Team provides various group programs that are focused on capacity-building in community and educational settings. Group work includes psychoeducation to assist community members to understand the mental health impacts of trauma. CLWs work alongside the CaB Team Coordinator and Counsellor Advocates to facilitate group programs as requested by services external to VFST. For example, requests may be made by schools that have a cohort of children from a particular community.

Community capacity-building work of CLWs is broad in its scope and is based on the needs of the community. This work is guided by the relationships established between CLWs and their respective communities. As outlined in “A Framework for Community Capacity Building” (2017) there are ten stages that underpin working with individual communities, covering identification of need, initial strategy development, consultation and development and review of the implementation plan:

1. Understand the key concerns of a refugee community
2. Establish a relationship with a refugee community
3. Develop and plan strategies to address a key concern for the refugee community
4. Identify people of influence in the refugee community to participate in the capacity-building process
5. Establish a structure including recruitment for an advisory group
6. Map assets to identify community goals
7. Form relationships with external service providers
8. Engage in dialogue towards identifying and achieving community goals
9. Extend reach of the outcomes to the whole community
10. Support community capacity-building sustainability [27, 28].

### Part 2: Qualitative study of the perspectives of CLWs

All seven CLW participated in the research. As can be seen from Table 1: demographic characteristics of CLWS, five of the CLWs had more than 11 years’ experience in their position. With regards to their cultural identity, three CLWs were Assyrian, two were Karen, one was Sierra Leonean and one was of Chin background.

**Table 1:** Demographic characteristics of CLWS

Variable	N
Duration of service in the role of CLW (years)	
1–5	2
6–10	0
11–15	5
Gender	
Female	2
Male	5
Age (years)	
20–34	1
35–49	1
50–64	3
Highest level of academic qualification	
Secondary school	1
Graduate	5
Postgraduate/doctoral	1
Language	
English	6
Krio	1
Hakha (Chin)	1
Burmese	3
Falam	1
Mizo	1
Karen	2
Assyrian	3
Arabic	3
Chaldean	2
Cultural identity	
Sierra Leonean	1
Chin	1
Karen	2
Assyrian	3

The following is a summary of the key themes identified on the basis of thematic analysis of the seven transcripts with CLWs (Table 2).

**Table 2:** Key themes regarding the work of CLWs in trauma-informed capacity-building strategies with refugee communities

1. The role of CLWs is diverse, multi-faceted and complex
2. CLWs enable members of refugee communities to access services and empower them to develop independence
3. CLWs enhance the capability of service providers
4. The community standing of CLWs supports their work in capacity-building
5. Goals of CLWs are focused on identifying and addressing the community’s areas of need, and working to promote the self-sufficiency of the community
6. The integration of perspectives is important to promote the long-term sustainability of capacity-building initiatives developed by CLWs
7. There is a need for allocation of adequate resources to focus on capacity-building
8. There is a need for broader recognition of the diverse and complex roles of CLWs
9. CLWs need to manage the expectations of community members
10. Peer support and supervision are integral to the work of CLWs

#### 1. The role of CLWs is diverse, multi-faceted and complex

A key theme that emerged was the diversity of responsibilities that characterises the role of CLWs. CLWs identified the management of flexibility inherent in their job.

Really (the role of a CLW) depends on the issues because of the flexibility and the changing nature of my job. (CLW-1)

All seven CLWs identified a key aspect of their role as providing a bridge between their community and service providers.

We always use the word “bridge” between communities and service providers. It’s an important role because we can identify issues, challenges, ways of engagement with the community. At the same time we can…let service providers know about the challenges and…barriers to accessing services and ways to…suit the community needs. (CLW-6)

A component of the complexity of the role of CLWs related to the various types of assistance that members of the community required of them in advocacy and in many aspects of the settlement process.

It’s…my passion…to…support the community by providing advocacy for the community. I’m providing advocacy in different levels… We identified…a group of overseas drivers…they didn’t have a full licence, so I advocated for their licenses…and we’ve been successful…they applied for truck licences, and they started working. (CLW-5)

As part of the diversity of their role, CLWs identified the service provided to individuals and their families attending VFST in joint work with Counsellor Advocates. CLWs also identified their role in various group activities, For example two CLWs identified their role in facilitating a therapeutic group, namely in collective healing, that uses a trauma-informed perspective to allow refugee community members to share their experiences.

Collective healing…We…formed the group ”Healing of memory”…We run…regular workshops inviting the refugee community…the context of the healing. We are talking about the past, present and the future… I am co-facilitating, also I have to share my story of trauma. Also within the group we support each other. (CLW-3)

CLWs also identified their role as encompassing the development and implementation of community initiatives that involved building the capacity of community leaders through community consultation, and creating a dialogue with management staff at VFST.

The community engagement…starts because the newly arrived community …are not familiar with the Foundation House system of counselling work. …We start doing that community consultation—we meet with them—we inform them about our services and…discuss with…leadership about how to engage them. (CLW-3)

#### 2. CLWs enable members of refugee communities to access services and empower them to develop independence

All CLWs identified that their position enables them to act as a resource for the community, and to enable members of refugee communities to not only access services but also to be empowered to develop independence in the long-term.

One CLW identified their impact as a resource person for the community to identify opportunities and services to meet particular unmet needs.

When we are here as refugees, there are so many opportunities that the refugee communities are not aware of and they do not know how to access it. So…as a CLW, I am…a resource person in the community to…point them to opportunities...whether for families, for the community as a whole. (CLW-1)

Another CLW identified that being in such a position enabled them to identify solutions for the community.

So our community get benefits from us….to find solutions or to provide support for the community. (CLW-5)

#### 3. CLWs enhance the capability of service providers

A core theme related to the role of CLWs in developing the capacity of services and service providers at many levels.

At a systemic level, one CLW gave insight regarding their role in improving services used by refugees.

A lot of service providers having developed their capacity…are well established and now have a lot of bicultural workers…providing settlement service, education and…employment service… (CLW-3)

Two CLWs identified their improvement of service providers’ cultural understanding of community needs as a key aspect of building this relationship, and integral to trauma-informed capacity building.

Service providers...they don’t really know the communities—their needs…So the work of the CLW is to bring the two parties together to understand each other, so that the service user can better target their services to better plan their services... (CLW-1)

#### 4. The community standing of CLWs supports their work in capacity-building

A key factor that was identified as a facilitator of the work of CLWs was their standing in the community as leaders, and their understanding of the community’s experience of trauma. This close relationship with members of the community was considered to be imperative in developing trust. One CLW identified that having a strong relationship with the community allows hidden issues to be identified and addressed.

I need to involve deeply with the community and make the relationship with the community very transparent…to be able to identify more issues…maybe we will be having hidden issues within the community that is destroying the community. (CLW-5)

#### 5. Goals of CLWs are focused on identifying and addressing the community’s areas of need and working to promote the developing self-sufficiency of the community

With regards to short-term goals identified by the CLWs, the overarching theme to emerge was that of the diversity of initiatives in trauma-informed community capacity-building that CLWs are engaged in, reflecting their various roles and the many different needs of their communities, including individuals marginalised within their communities. For example one CLW identified their goal to empower men who may face marginalisation in order to help them find purposeful activities.

The men in our culture…they feel themselves…they don’t have any meaning for their life. So I’m interested to establish this group…to empower them…because they were powerful…in their country of origin…regain their capacity and their capability. (CLW-5)

Another CLW identified their goal in addressing marginalisation of young people through reconciliation of intergenerational conflict in their community, arising as a result of the experience of trauma by the community as a whole.

There are issues between the young people and their community elders. So that is a subject of ongoing intervention. We want to see how to bring the two parties together. (CLW-1)

With respect to the long-term goals of CLWs in their role of trauma-informed capacity-building the development of self-sufficiency within their communities was identified as of great importance. Two CLWs articulated this goal of ensuring the sustainability of the work of community groups so that they may become self-sufficient, hence making the CLW role redundant.

My desire…is for me to be redundant, so that there won’t be any need for me. If that can happen then I can say we’re successful…nobody needs me because they have been resourced enough to do things without me. (CLW-1)

#### 6. The integration of perspectives is important to promote the long-term sustainability of capacity-building initiatives developed by CLWs

CLWs identified particular challenges in integrating diverse perspectives in the long-term, particularly when there may be competing agendas. The challenges in managing diverse demands from different groups within their community was noted.

Intergenerational gap…for our next generation it’s going to be a challenging process for them to maintain…the two different cultural identity differences. Their parents…still want to keep their own identity…For younger people or children who are born here…to have a kind of compromise to adapt in both ways…can keep the community healthy… a better way in the integration process. (CLW-2)

As expressed by one CLW, at times the integration of perspectives from external community experts may not be sustainable unless they are relevant to the community. Reaching a consensus was considered to be important to the sustainability of community projects.

Sometimes professional persons not from the…community…it doesn’t work because we have many strategies, many theories that could be implemented…But many of them they don’t work with that community because we have many ethnicities… In my opinion, we need both…we need professional persons and we need persons from the community…to understand…what strategies…work with the community…what don’t work (CLW-5)

#### 7. There is a need for allocation of adequate resources to focus on capacity-building

Four CLWs identified the lack of secure external funding for community projects as a significant challenge to their work in capacity building.

One of the bases of getting funding for our communities is through…grants. So if our community does not get grants from councils or state government than we don’t have anything. And so that undermines my ability to organise and run programs… (CLW-1)

The lack of secure financial resources was noted to be a barrier to the sustainability of projects, and two CLWs advocated for a sustainable long-term funding model to provide a greater level of security for specific programs, so that they are not discontinued.

Sometime we have…one-off funding so… it’s good for at least…two or three years…Instead of one-off project maybe…long-term…the community will take over….this will be more sustainable… Nowadays one project does not have the funding for another two or three years again… we start again so there’s not development of capacity... (CLW-3)

#### 8. There is a need for broader recognition of the diverse and complex roles of CLWs

With regard to strategies to enhance the role of CLWs, the need to formally recognise the complexity within the role of CLWs, including the dual nature of their work as employees of VFST and also community leaders was identified. The CLWs identified that their dual role may create conflict unless formally recognised.

We have two roles…community leader and…CLW. When we approach our community…we are approaching them as community leaders. And…at Foundation House we approach them as CLW…so still the community are looking at us as community leaders. So it depends on Foundation House to…understand that relationship…and…to provide us with that sense of leadership…to provide us with support to maintain the trust of the community… We have capability to take initiative and to start working with the community within the groups…but we need…our organisation to provide us professional support. (CLW-5)

The burden of responsibility in being a representative of their community was also acknowledged. One CLW expressed insight into the significance of the responsibility involved in advocating and representing the community’s interests.

It’s being representative of the community…you are taking big responsibility and talking on behalf of the community which isn’t easy because it’s a big community and with the groups…sometimes….one group will agree with you but the other group won’t agree with you...so this is a challenge. (CLW-6)

Whilst diversity within their roles was recognised as a strength, potential ambiguities were also recognised to be problematic. The potential benefit of guidelines to assist CLW activities was identified as a strategy to address the complexity in their roles.

We should be having guidelines within our organisation…like…we have these issues…we have these activities…we have these plans…What do you suggest for us to do at this stage? (CLW-5)

#### 9. CLWs need to manage the expectations of community members

CLWs identified barriers to their work in managing the expectations of community members. Three CLWs identified that engagement in community activities may at times go beyond the scope of their role at VFST, which can be personally demanding.

The first challenge is…role clarification…so we are part of the community and also part of the professional organisation…sometimes our role is not clear… Sometimes…we are working on the weekend… community engagement…which part of the activity is core of our Foundation House work and which part of the activity is the community work? (CLW-3)

The challenges involved in negotiating cultural differences between members of the community and the organisation also emerged.

The communities…practice different… in traditional ways. Sometimes we will have to work according to the structure here….Quite a…challenge…to take a community member or working group voice and also to follow the structure here…it’s sometimes a challenge between those two systems. (CLW-4)

Five CLWs identified the challenge of coping with requests for support from the community outside working hours.

As a community member people always call us even after hours…even if it’s not related to the service that we provide…they think that we know about all the service providers and to direct them…And you can’t say no because the expectation is that you would be there for the community whenever they need you. (CLW-6)

Two CLWs identified that because of the demanding nature of their work, they lacked time for self-care.

When we are working it’s a very challenging situation…we didn’t have enough time to rest ourselves. (CLW-3)

Three CLWs expressed insight into the dilemmas involved in identifying the limits of the level of support that may be offered, particularly considering that members of the community they serve are themselves survivors of trauma. Managing the high expectations of community members was noted to be a particular challenge.

Community has very high expectation from us. The reality is that we can give…services that are trauma [informed]…The community are expecting everything from us… they are expecting we are experts. The other thing is culturally you can’t say no sometimes but reality is we work with Foundation House so sometimes we have to say no. (CLW-3)

#### 10. Peer support and supervision are integral to the work of CLWs

The value of peer support from members of the CaB Team was identified. All CLWs identified the value of training and professional development, as well as the formal peer support process involving CLWs and the CaB Team Coordinator.

We receive a lot of training…So always we do a lot of professional development in different ways…The supervision we do weekly as well, that helps us very much (CLW-7)

Team peer support is very good …we have other CLWs so we can talk anytime if you think you need help. Sometimes we need the extra support. (CLW-3)

Three CLWs identified that the support of peers allowed CLWs to share information and knowledge readily, so that initiatives found to be effective with one particular community could be shared with others.

They already have a lot of experience with their work. If I face any challenges or problems in the community dealing with a particular issue….asking them…whether they have experienced anything similar to my experience…we discuss everything openly…The community also benefits from other communities as well. So sharing information and knowledge helps us a lot. (CLW-2)

CLWs particularly identified the importance of supervision in order to cope with trauma that they may be exposed to vicariously through their work.

I’m working with professionals who are dedicated specialists within this sector. So I receive support from them…how to deal with the impact of trauma, how to protect myself… (CLW-5)

## Discussion

This study sought to review the establishment and evolution of the VFST CLW role, to describe the role of CLWs, and to examine the perspectives of CLWs on their role in trauma-informed community capacity-building.

This study provides an insight into the nature, diversity and complexity of the work of CLWs. The CLWs articulated that their bridging role encompasses the provision of advocacy, networking, empowerment of community members and cultural orientation for service providers. Similar to Baile’s report [6], CLWs were identified as acting as facilitators to assist refugee communities in accessing services and fostering linkages between members of the community. This study also contributes to the literature regarding the importance of the role of liaison workers as cultural consultants, even though the VFST CLW role has shifted away from its emphasis on this aspect of work in order to engage in more broadly defined trauma-informed capacity-building interventions [10, 19, 40]. This study also confirms the importance of the liaison worker as being uniquely qualified to provide an advocacy role and to mediate conflict [16, 20, 41].

As described by Masinda, the concept of enhancing immigrant settlement service literacy can be used to describe the process whereby individuals understand and are able to navigate mainstream services [42]. The empowerment of refugee communities to gain skills to effectively mobilise the mainstream society so that relevant services are relevant to their communities and are an important part of the broader social agenda is inherent in the role of capacity building initiatives such as the CLW role [42].

Being a resource for their community and working to improve the cultural responsiveness of service providers were identified as beneficial impacts for refugee communities of engaging CLWs in this study. McBrien and Ford [9] also demonstrated the positive benefits of engaging refugee parents as liaison workers to support children at school [9]. As noted in this study, the CLW role is broad and not confined to a specific role or project. The CLWs in this study noted the impact of various interventions to address the needs of at-risk groups, including youth members of their respective communities. Similarly, Mortensen et al. [15] demonstrated the positive contribution liaison workers were able to make in improving access to child health and disability services by fostering the relationship between service providers and the community as well as improving the cultural responsiveness of services [15].

The CLWs identified numerous facilitators of their work. Trauma-informed interventions of any kind are required to have long-term aims, reflecting the potentially enduring nature of trauma impacts. Capacity building initiatives with refugee communities must be tailored to meet the needs of the community members themselves and be implemented through trauma-informed processes which emphasise active, participatory functions [1]. CLWs in this study incorporated a trauma-informed understanding of issues in developing strategies particularly in terms of establishing safety and trust. Similar to other studies, the work of CLWs in trauma-informed capacity-building initiatives is facilitated by their shared identity and ethnicity [6, 23, 43], shared refugee experience [10, 16, 26] and skills [10, 40, 44]. CLWs in this study engaged in many similar approaches to the work of liaison workers reported in the literature, including strategies to improve the cultural appropriateness of services [20, 22, 45], the use of peer review and support [46, 47], emphasis on the development of trusting relationships between refugees and service providers [13, 23, 48], adopting a holistic needs-based approach [46, 49, 50] and developing various group programs in order to address the needs of the community [8, 45].

At the same time, a number of challenges were identified by CLWs in the work of capacity-building. The availability of external funding for projects was recognised as a facilitator of the work of capacity-building undertaken by CLWs, although the lack of a secure source of funding was recognised as a challenge, and a barrier to the sustainability of community initiatives. Furthermore, whilst the diversity of responsibilities is an integral aspect and strength of the CLW role, this also poses challenges. In particular, because of the breadth of their community engagement work, many CLWs provide such support to their community outside working hours, that was noted to be personally demanding. The need for clarity in their roles as employees of VFST was recognised. CLWs are also required to manage the high expectations associated with the position of community leader. In addition, the liaison role that CLWs perform inevitably places them in a position where their task may require them to recognise and address discrepancies between the viewpoints of community members and service providers. Other studies have also highlighted the need for appropriate boundaries given the limited availability of liaison workers [20], the potential for over-involvement with clients [51], the limited evidence-base regarding capacity-building interventions in refugee communities [17, 52] and challenges in time management [23, 45]. This study also supports the literature regarding the challenges that liaison workers in general face with regards to reconciling differences in cultural perceptions regarding health [46, 53] and managing expectations from both the community and service providers [47, 54]. Furthermore, this study confirms previously reported findings regarding the need to ensure the personal wellbeing of liaison workers given the demanding nature of their work [40, 46, 55].

The strengths of this study are that it sought to understand the perspectives of liaison workers themselves regarding the facilitators and barriers to their work with their respective communities. This study has contributed to the literature regarding the role of liaison workers of refugee background in identifying and addressing the priorities of their communities as well as improving understanding and awareness amongst mainstream and specialist services. By adopting a qualitative design, an in-depth exploration of the perspectives of a small group of liaison workers was possible, and further research may build on this understanding of their role in trauma-informed capacity building.

The researchers acknowledge several limitations of this study. First, CLW participants were recruited only from VFST. Hence the study findings may not be generalizable to other settings. However, it is anticipated that the findings of this study will be of relevance to services working with refugee communities. Second, the study involved a small number of participants. Previously employed CLWs were not engaged in the study, as the purpose of the research was to gain insights from the current members of the CaB Team. Third, the study was conducted at a single point in time. The Case study section of the manuscript outlines a retrospective view of the history and evolution of the CLW role in the context of the CaB Team. It was not feasible to utilise a prospective study design using ethnographic research methods in order to document the evolution of the CLW role in more detail. Fourth, the study provides an insight into the perspectives of CLWs based on their experience across several years of working with their communities. A formal evaluation of particular capacity-building projects involving CLWs to examine the impact of their role was not conducted. Further research work that focuses on the CLW role within specific initiatives is warranted. Fifth, the study focused only on the views of CLWs themselves. The views of other stakeholders including members of the community and other VFST staff were not sought for the purpose of this study. Triangulation methods to assess the viewpoints of other stakeholders may enhance the validity of this study’s findings.

## Conclusions

This qualitative study provides insights into the diverse role of the CLW at VFST, a service for refugees in Victoria, Australia. An essential feature of the CLW role is that members of refugee communities are engaged to work with their respective communities in order to improve access to services and to provide trauma-informed capacity-building initiatives to meet their communities’ particular needs. The CLWs adopt a trauma-informed perspective in their work, supported by the organisation, which is characterised by the development of trust, collaboration with other stakeholders, empowerment, and building on the strengths of refugee communities who have collectively experienced trauma. CLWs see clear benefits to their communities through their work within VFST and their ability to enhance engagement between service providers and their communities. CLWs face numerous challenges in managing community expectations, prioritising areas of need and personal challenges arising from the demanding nature of the work. Sustainability of trauma-informed capacity-building initiatives is dependent on the strengthening of the relationship between community members and service providers, facilitated by CLWs. The CLW role and the work of the CaB Team at VFST continue to evolve and develop in response to the needs of refugee communities. Further research is required to evaluate the impact of specific capacity-building projects in which CLWs are involved, and to understand the perspectives of community members and other stakeholders regarding the impact of the CLW role.

## Data Availability

The datasets used and/or analysed during the current study are available from the corresponding author on reasonable request.

## Abbreviations

VFST: Victorian Foundation for Survivors of Torture
CaB Team: Capacity building team
CLW: Community liaison worker
